# Supplementary figures and images for: Chromatin organization changes during the establishment and maintenance of the postmitotic state
Source: Epigenetics Chromatin. 2017 Nov 10;10:53. doi: 10.1186/s13072-017-0159-8 (PMC5681785; doi:10.1186/s13072-017-0159-8)

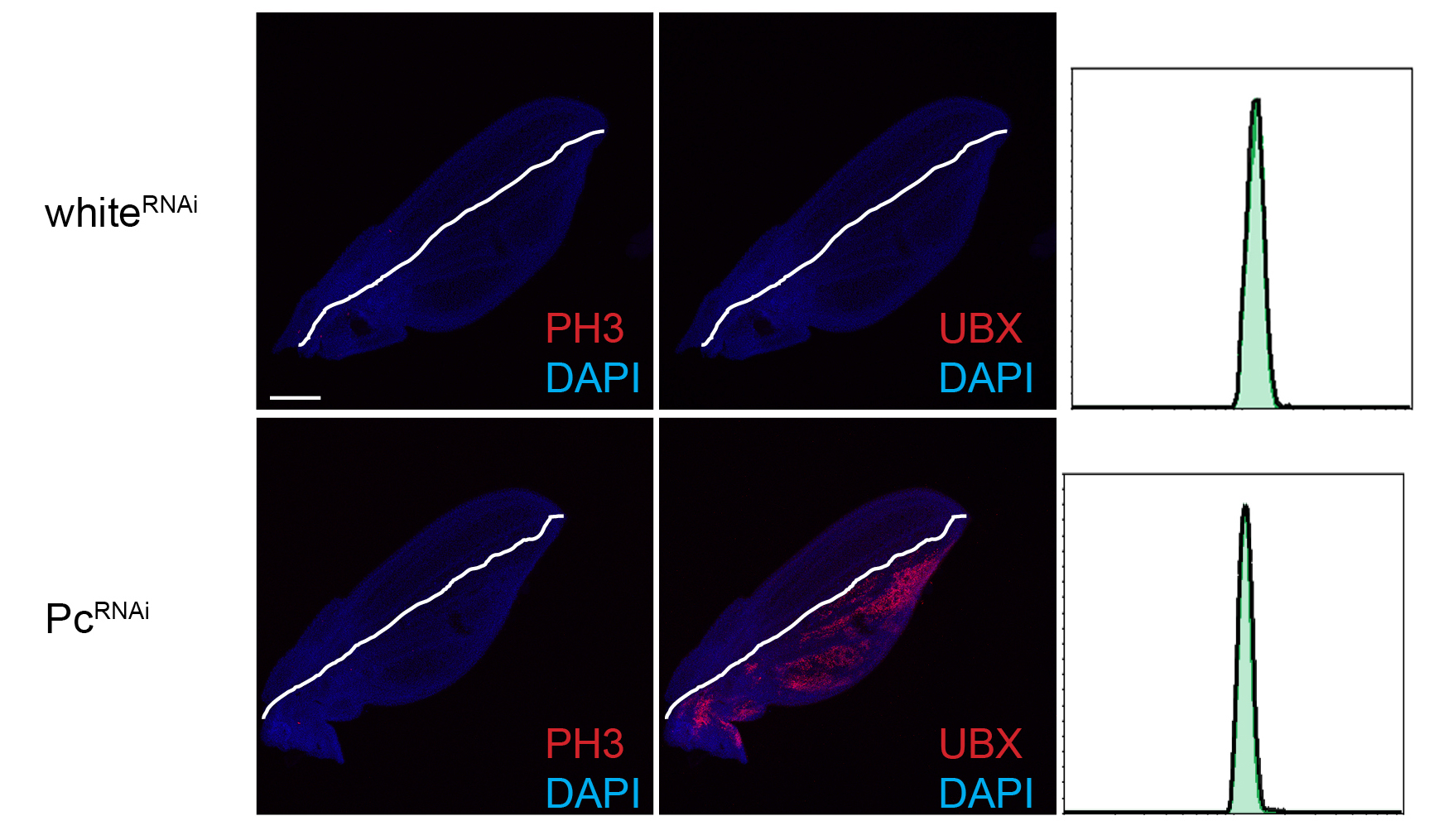

Supplement: Supplementary file 1 — Additional file 1: Figure S2. Compromising PRC1 does not delay cell cycle exit. RNAi to Pc or white (as a control) was expressed in the posterior wing from the L3 stage and postmitotic wings at 26–28 h were examined for mitoses as indicated by PH3 and effective knockdown of PRC1 function by de-repression of the PRC1 target gene Ubx. Flow cytometry was also performed to measure cells that enter S and G2 phases. Green trace indicates cells from the posterior wing expressing the indicated transgenes. Black trace: control non-expressing anterior wing cells. Compromising PRC1 activity does not delay cell cycle exit. Scale bars = 100 μm. [file 13072_2017_159_MOESM1_ESM.jpg]

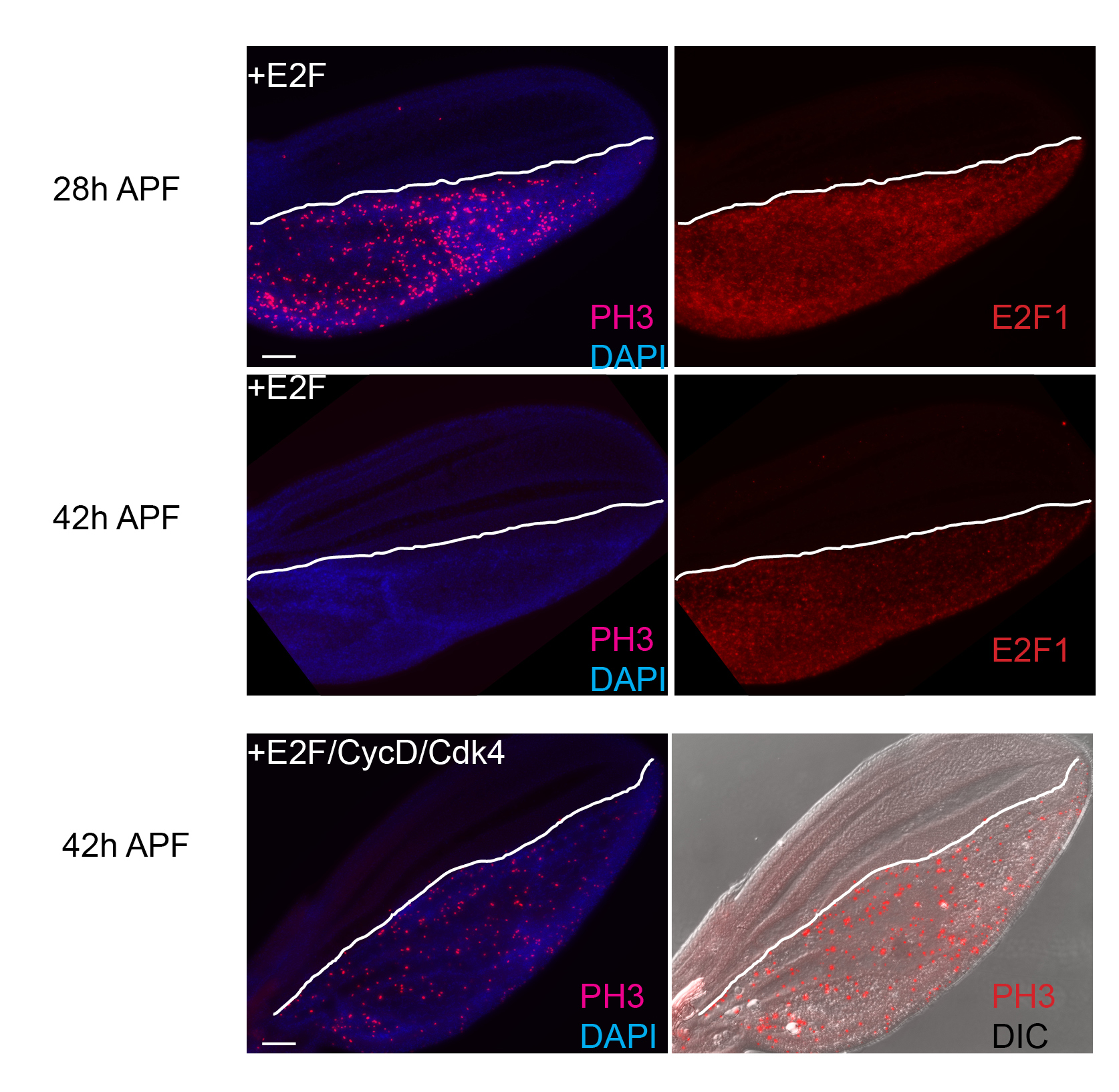

Supplement: Supplementary file 2 — Additional file 2: Figure S3. Two stages of G0 in differentiating wings. E2F was expressed in the posterior wing to delay cell cycle exit. 28 h and 42 h APF pupal tissues were dissected and immunostained for PH3 (to label mitoses) and E2F1. The anterior/posterior boundary is specified by the white line. Overexpression of E2F delays entry into G0 until 36 h. At 42 h cells expressing high E2F1 are postmitotic (in robust G0). CycD/Cdk4 + E2F expression in the posterior wing is able to bypass the robust G0 to promote continued cycling, as shown by abundant mitoses (PH3) at 42 h. Bar = 50 μm. [file 13072_2017_159_MOESM2_ESM.jpg]

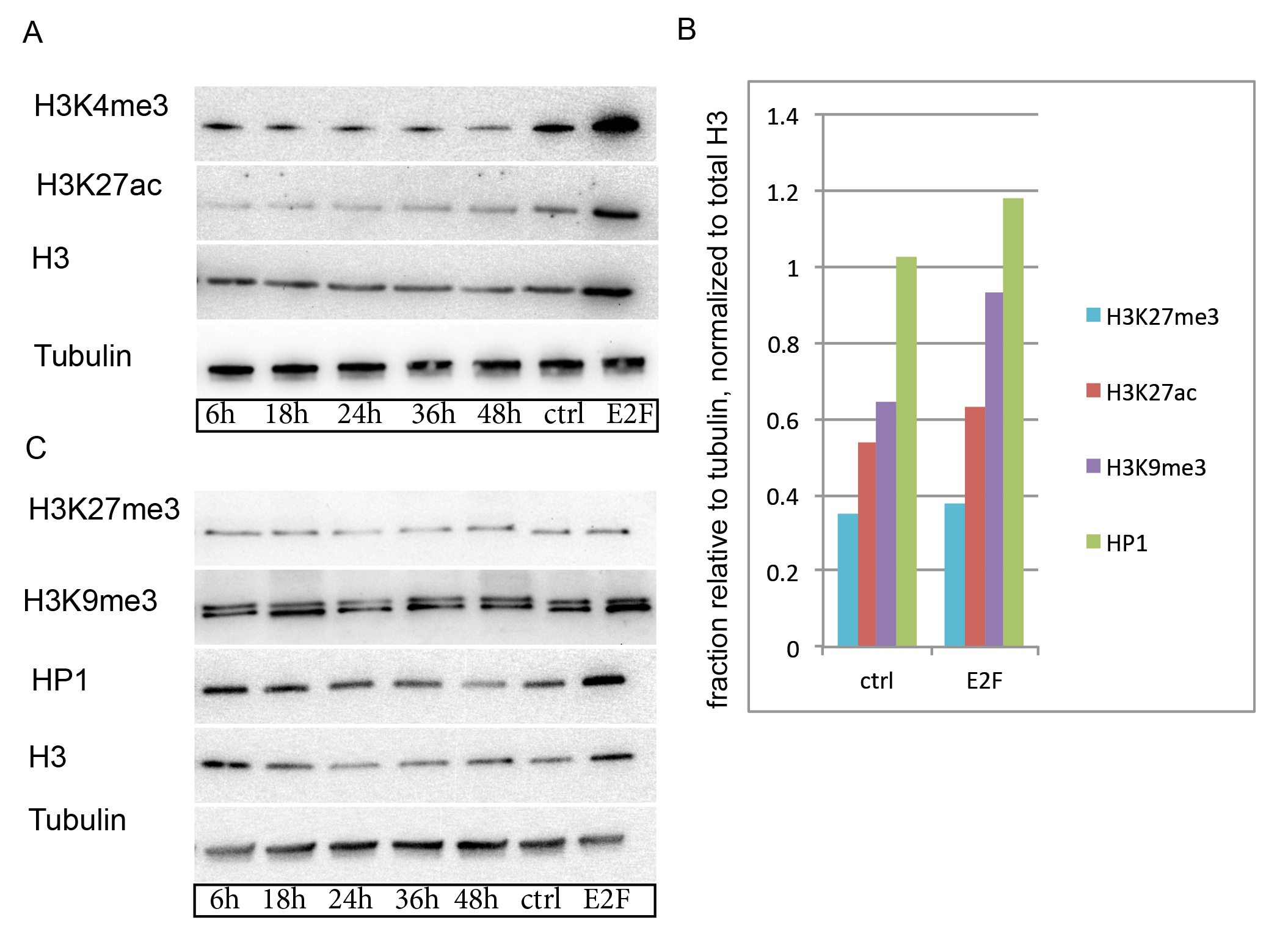

Supplement: Supplementary file 3 — Additional file 3: Figure S1. Global levels of histone modifications do not dramatically change at cell cycle exit. (A-D) Quantitative western blots were performed on wings of the indicated stages to assess the levels of modified or total histone H3 or HP1. Control (Ctrl) and E2F samples are from 28 h postmitotic wings overexpressing GFP or E2F respectively. Total H3K9Me3, H3K27Me3, and HP1 levels do not dramatically change with cell cycle exit, however they increase with E2F expression. Modifications associated with active chromatin, H3K4Me3 and H3K27Ac also do not dramatically change with cell cycle exit, but increase upon E2F expression. [file 13072_2017_159_MOESM3_ESM.jpg]

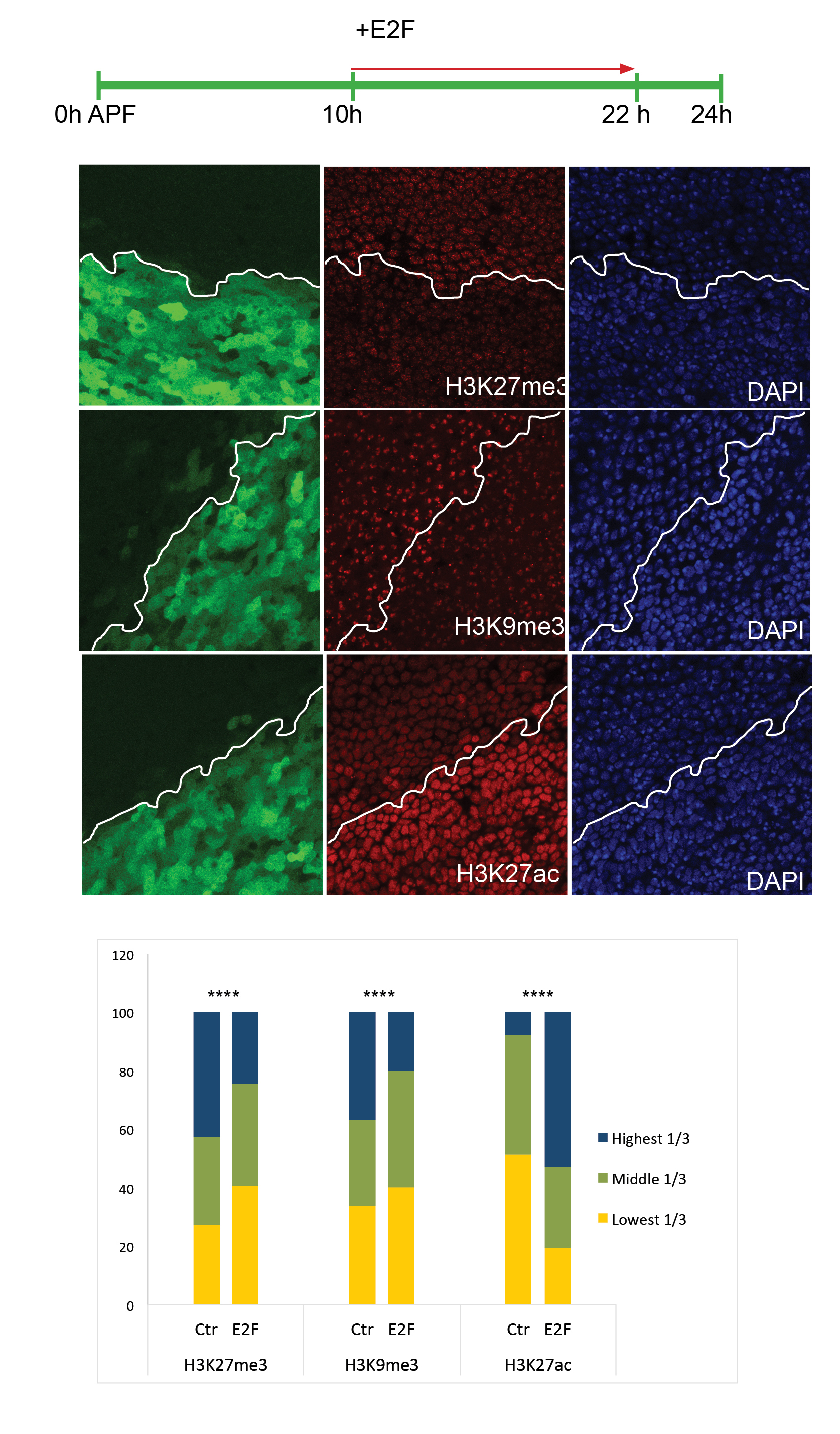

Supplement: Supplementary file 5 — Additional file 5: Figure S4. Clustering of heterochromatin can be disrupted within one cell cycle. E2F was overexpressed in the posterior wing from 10 h APF. 12 h later (within approximately one cell cycle) tissues were immunostained for indicated histone modifications. The posterior region is labeled by the expression of GFP and the anterior/posterior boundary is specified by the white line. The distribution of staining intensity in 1112–1339 nuclei, binned into three ranges, is shown at bottom. E2F disrupts heterochromatin clustering within one cell cycle. P values were determined by an unpaired t test; ****< 0.0001. [file 13072_2017_159_MOESM5_ESM.jpg]

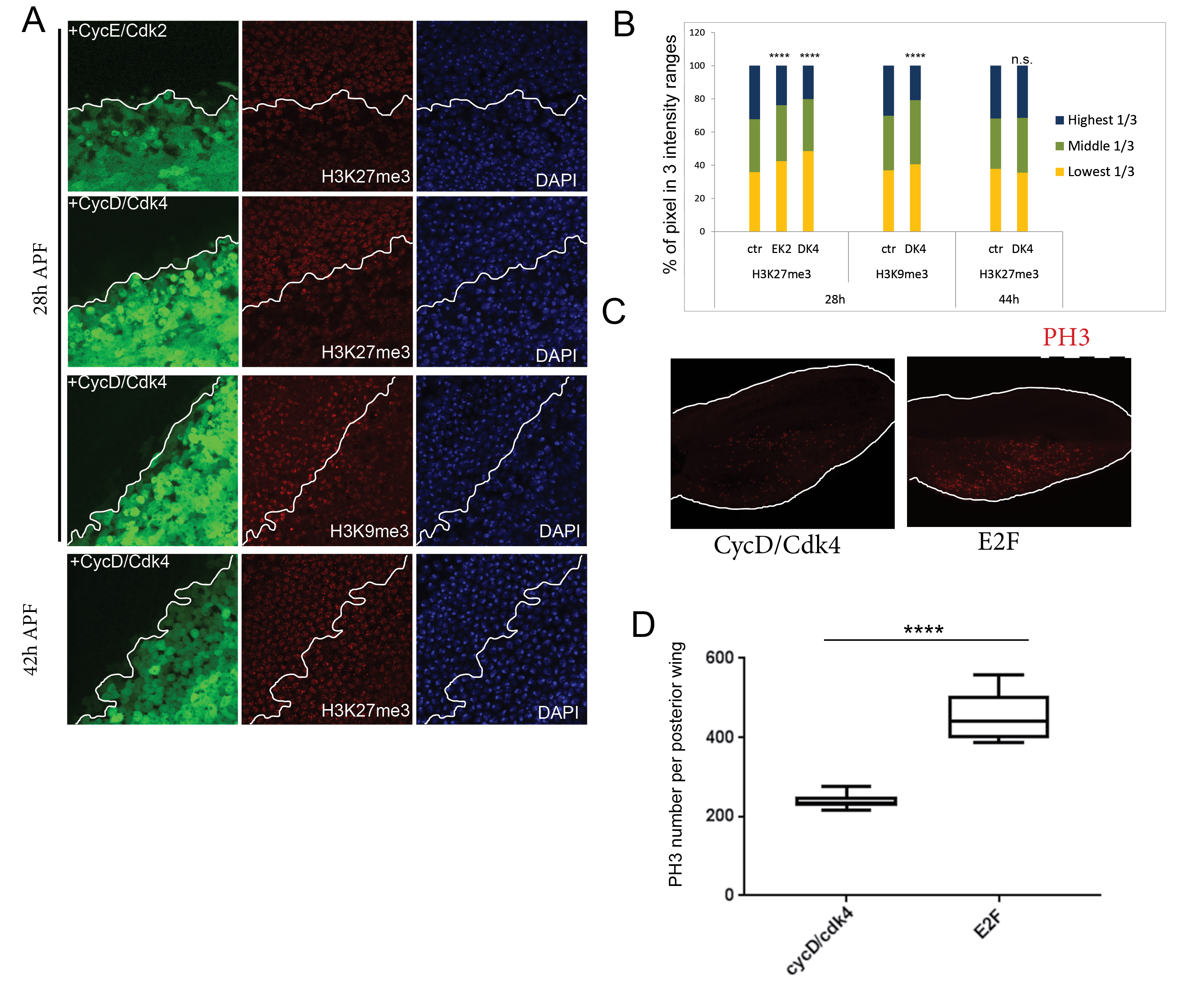

Supplement: Supplementary file 6 — Additional file 6: Figure S5. Delaying cell cycle exit disrupts heterochromatin. (A) CycE/Cdk2 or CycD/Cdk4 complexes were overexpressed in the posterior wing from 0 h APF. The anterior/posterior boundary is indicated by the white line. At 28 h (flexible G0) or 42 h APF (robust G0) pupal tissues were dissected and immunostained for the indicated histone modifications. (B) The distribution of staining intensity from 492 to 976 nuclei, binned into three ranges, is shown. Wings expressing E2F or CycD/Cdk4 to delay cell cycle exit were stained for mitoses (PH3) and the mitotic index at 27 h was quantified for the posterior compartment (C-D). The degree of heterochromatin disruption correlates with the number of cells cycling. P-values were determined by an unpaired t test; ****P value < 0.0001. [file 13072_2017_159_MOESM6_ESM.jpg]
