# Supplementary material for: Chromatin organization changes during the establishment and maintenance of the postmitotic state
Source: Epigenetics Chromatin. 2017 Nov 10;10:53. doi: 10.1186/s13072-017-0159-8 (PMC5681785; doi:10.1186/s13072-017-0159-8)
Supplement: Supplementary file 4 — Additional file 4: Table S1. Chromatin modifiers/organizers/remodelers that are upregulated upon E2F1/DP expression in pupal wings. [file 13072_2017_159_MOESM4_ESM.docx]

**Supplemental Tables**

Supplemental Table 1.

Chromatin modifiers/organizers/remodelers that are upregulated upon E2F1/DP expression in pupal wings

| Gene | Function | log_2_FC | Adj. p. value |
| --- | --- | --- | --- |
|  | Histone Biosynth. /Nucleosome assembly |  |  |
| *Slbp* |  | 3.01 | <0.001 |
| *mxc (NPAT)* |  | 0.58 | <0.001 |
| *Lsm10* |  | 2.70 | <0.001 |
| *Cpsf* |  | 1.80 | <0.001 |
| *Asf1* |  | 2.09 | <0.001 |
| *hd* |  | 3.54 | <0.001 |
| *Caf1-105* |  | 2.87 | <0.001 |
| *Caf1-180* |  | 2.35 | <0.001 |
|  | CHRAC complex |  |  |
| *Acf1* |  | 1.55 | <0.001 |
| *ISWI* |  | 1.07 | <0.001 |
| *Chrac-14* |  | 2.73 | <0.001 |
|  | NuA4/TINTIN complexes |  |  |
| *Reptin* |  | 1.55 | <0.001 |
| *domino* |  | 0.50 | <0.001 |
| *Mrg15* |  | 0.66 | <0.001 |
| *dMap1* |  | 0.58 | <0.001 |
| *CG2982* |  | 1.55 | <0.001 |
|  | Trithorax/TRR complexes |  |  |
| *Pa1* |  | 1.35 | 0.0039 |
| *Mnn1* |  | 1.40 | <0.001 |
| *ash1* |  | 0.65 | 0.0166 |
| *Ptip* |  | 1.74 | <0.001 |
| *CG33695 (Bap18)* |  | 1.69 | <0.001 |
| *trr* |  | 0.81 | 0.0025 |
|  | NSL/MSL complex |  |  |
| *mof* | H4K16 acetylase | 2.35 | <0.001 |
| *msl-1* |  | 0.68 | 0.0200 |
| *MBD-R2* |  | 0.81 | <0.001 |
|  | DREAM/MMB complex |  |  |
| *mip120* |  | 2.51 | <0.001 |
| *Rbf* |  | 2.24 | <0.001 |
| *lin-52* |  | 1.18 | 0.010 |
| *E2f2* |  | 0.74 | <0.001 |
|  | Insulators |  |  |
| *CG9740 (Ibf2)* |  | 1.11 | <0.001 |
| *BEAF-32* |  | 1.67 | <0.001 |
| *pita* |  | 1.53 | <0.001 |
| *DREF* |  | 1.25 | <0.001 |
|  | NuRD complex |  |  |
| *Mta1-like* |  | 0.78 | <0.001 |
| *rpd3* | H3K27 deacetylase | 0.91 | <0.001 |
| *Chd3* |  | 4.08 | <0.001 |
|  | PBAF complex |  |  |
| *Bap170* |  | 1.85 | <0.001 |
| *Polybromo* |  | 1.61 | <0.001 |
| *Bap55* |  | 1.10 | <0.001 |
| *Bap111* |  | 0.74 | <0.001 |
|  | PRC2 complex |  |  |
| *esc* |  | 3.61 | <0.001 |
| *Su(z)12* |  | 0.97 | <0.001 |
| *Pcl* |  | 0.89 | <0.001 |
| *E(z)* | H3K27 methyltransferase | 0.88 | <0.001 |
|  | PhoRC Complex |  |  |
| *phol* |  | 0.95 | <0.001 |
| *sfmbt* |  | 0.90 | <0.001 |
|  | HP1 complexes |  |  |
| *Su(var)3-9* | H3K9 methyltransferase | 0.95 | <0.001 |
| *HipHop* |  | 3.78 | <0.001 |
| *cav (HOAP)* |  | 2.02 | <0.001 |
| *Rif1* |  | 2.63 | <0.001 |
| *Mes-4 (NSD)* |  | 1.75 | <0.001 |
| *HP5* |  | 1.05 | <0.001 |
| *Su(var)2-HP2* |  | 1.29 | <0.001 |
|  | Other chromatin regulators |  |  |
| *SuUR* |  | 1.75 | <0.001 |
| *PR-Set-7 (Set8)* | H4K20 methylase | 1.42 | <0.001 |
