# Supplementary material for: Chromatin organization changes during the establishment and maintenance of the postmitotic state
Source: Epigenetics Chromatin. 2017 Nov 10;10:53. doi: 10.1186/s13072-017-0159-8 (PMC5681785; doi:10.1186/s13072-017-0159-8)
Supplement: Supplementary file 7 — Additional file 7: Table S2. Genes associated with senescence that are upregulated during robust G0 in the presence of ectopic E2F1/DP. [file 13072_2017_159_MOESM7_ESM.docx]

Supplemental Table 2

Genes associated with senescence that are upregulated during robust G0 in the presence of ectopic E2F1/DP.

| Gene | Possible mammalian ortholog | | log_2_FC | Adj. p. value |
| --- | --- | --- | --- | --- |
|  | SASP-phenotype | |  |  |
| *Mmp1* | Matrix Metalloproteinases | | 2.52 | <0.001 |
| *Ast-C* | CXCR2 ligand | | 2.51 | <0.001 |
| *Dsp1* | HMGB1 (SASP regulator) | | 0.58 | <0.002 |
|  |  | |  |  |
|  | NfkB/MapK/TGF-β signaling | |  |  |
| *p38c* | MAPKinase | | 1.38 | <0.001 |
| *pipe* | uronyl 2-sulfotransferase (UST) | | 0.73 | <0.002 |
| *Traf4* | TRAF4 | | 0.84 | <0.001 |
| *spitz* | TNFRSF1A associated via death domain | | 1.01 | <0.001 |
| *cv-2* | BMPER | | 1.17 | <0.001 |
| *shifted* | WIF1 | | 1.15 | <0.001 |
| *fog* | NECAP2 | | 1.36 | <0.001 |
| *CG4325* | TRAIP | | 0.87 | <0.002 |
| *frazzled* | DCC | | 0.82 | <0.001 |
|  |  | |  |  |
|  | Oxidative Stress | |  |  |
| *CG7737* | SMOX/KDM1B | | 0.92 | <0.006 |
| *CG31937* | dehydrogenase/reductase 7 | | 0.68 | <0.002 |
| *Nmdmc* | MTHFD1 | | 0.66 | <0.001 |
| *CG11200* | *DHRSX* | | 0.85 | <0.001 |
| *CG5599* | DBT | | 1.09 | <0.001 |
| *TPC1* | Thiamine pyrophosphate carrier 1 | | 1.52 | <0.001 |
| **Multiple Cytochrome P450 enzymes**  **Multiple DNA damage response genes** | |  |  |  |
|  |  | |  |  |
